# A prominent role of Hepatitis D Virus in liver cancers documented in Central Africa

**DOI:** 10.1186/s12879-016-1992-2

**Published:** 2016-11-07

**Authors:** Marie Atsama Amougou, Dominique Noah Noah, Paul Fewou Moundipa, Pascal Pineau, Richard Njouom

**Affiliations:** 1 Virology Unit, Centre Pasteur of Cameroon, BP 1274 Yaounde, Cameroon; 2 Central Hospital of Yaounde, Yaounde, Cameroon; 3 Laboratory of Pharmacology and Toxicology of University of Yaounde I, Yaounde, Cameroon; 4 Unité « Organisation nucléaire et Oncogenèse », INSERM U993, Institut Pasteur, Paris, France

**Keywords:** Hepatocellular carcinoma, Hepatitis B virus, Hepatitis C virus, Hepatitis D virus, Cameroon

## Abstract

**Background:**

Hepatocellular Carcinoma (HCC) is one of the commonest cancers in Central Africa, a region with the unusual peculiarity to be hyperendemic for infections with Hepatitis B, C and D viruses. However, data estimating the respective proportions of HCC cases attributable to these viruses are still limited in this area. The current study was undertaken to determine the role of these viruses in HCC compared to non-HCC Cameroonian patients.

**Methods:**

A case–control study was conducted in the Gastroenterology Unit of Central Hospital of Yaounde in collaboration with Centre Pasteur of Cameroon. Blood samples of all HCC cases (*n* = 88) and matched control individuals without known liver disease (*n* = 85) were tested for serological markers of Hepatitis B, C and D viral infections using commercially available enzyme immune-assay kits. Hepatitis B and C viral loads were quantified for positive patients by real-time PCR using commercial kits.

**Results:**

The mean age was 46.0 ± 18 and 42.1 ± 16 years old for HCC-patients and controls, respectively for a 2.3 Male/Female sex ratio. The prevalence of hepatitis B surface antigen, antibody to HCV and antibody to HDV were significantly higher in HCC patients (65.90, 20.26 and 26 % respectively) than in control patients (9.23, 4.62 and 1 %) (*P* < 2.5 10^−5^). The risk factors analysis showed that both HBV and HCV infections were strongly associated with HCC development in Cameroon with crude odds ratios of 15.98 (95 % CI 6.19-41.25) and 7.33 (95 % CI 2.09-25.77), respectively. Furthermore, the risk of developing HCC increased even more significantly in case of HBV and HDV co-infections with the odd ratio of 29.3 (95 % CI, 4.1-1231). HBV-DNA level was significantly higher in HBsAg-positive HCC-patients than in HBsAg-positive controls with (6.3 Log IU/mL and 5.7 Log IU/mL) respectively (*P* < 0.05).

**Conclusion:**

HBV and HCV infections are the mains factors of HCC development in Cameroon. Our results show that patients co-infected with HDV are at very high risk to develop HCC. An active surveillance program of patients and, foremost, an easier access to antivirals and primary prevention measures are crucial steps to reduce the incidence of HCC in this country. Due to the lack of truly efficient antiviral therapy, the fate of HDV-infected patients remains, however, particularly worrying.

## Background

Hepatocellular carcinoma (HCC) is the second most common cause of cancer death in the world and is particularly prevalent in Africa and Asia [1–3]. There is a strong correlation between the incidence of HCC and the prevalence of chronic hepatitis B and C, indicating that these two viral infections are some of the most important risk factors of HCC worldwide with a combined attributable fraction of at least 75 % of all HCC cases [4, 5].

Growing evidence indicates that the high incidence areas of HCC correspond primarily to the zones where chronic hepatitis B is prevalent and exposure to aflatoxin B1 (AFB1), a mutagenic mycotoxin, is frequent [6]. In addition to viral hepatitis infections and dietary AFB1 exposure many other risk factors associated with HCC are well documented. They include heavy alcohol consumption, iron overload, type II diabetes and cigarette smoking [6].

In Cameroon, HCC has been reported as the country’s commonest type of cancer [7]. Cameroon, as other countries of Central Africa (*eg* Burundi) is also a hyper-endemic area for hepatitis B, C and D viral infections with HBsAg prevalence ranging between 6 and 33 % [8–11], anti-HCV between 4 and 30 % [11, 12] and anti-HDV between 13 and 62.5 % of HBV surface antigen (HBsAg) carriers [13] depending on the population and the area studied.

However, like for other developing countries in Central Africa, recent and first-hand data regarding the involvement of hepatitis B and C viruses in HCC is rather limited [14]. The few available studies carried out in Cameroon bring out essentially the clinical, epidemiological and diagnostic aspects of HCC resulting from cross sectional analyses [15, 16]. So far, information on the respective involvement of HBV and HCV in HCC remains unknown. The current study was undertaken to assess the risk associated with the three viruses in HCC-cases compared to HCC-control (non-hepatic disease) Cameroonian patients using a case–control study.

## Methods

### Patients

A case–control study was performed. Cases were made of HCC patients consecutively enrolled in the Gastroenterology and Radiology Units of Central Hospital, Yaoundé between February 2013 and January 2014. They were individually 1:1 paired-matched by sex and age (±5 years) with control subjects consecutively selected and represented by patients without clinical symptom of liver diseases attending at the same period the same medical departments.

The diagnosis of HCC was based on presence of a liver mass at ultrasound and, when possible, histology of tissues samples together with elevation of serum alpha-fetoprotein (AFP) (>400 ng/ml) levels. Of the 88 HCC cases included, 61 % (*n* = 54) were found with AFP levels of 400 ng/mL or higher, 20.5 % (*n* = 18) had AFP level less than 400 ng/mL or above normal values (>16.5 ng/mL). In the remaining cases (*n* = 16) AFP were found normal (<16.5 ng/mL). Inclusion criteria for control subjects without clinical evidence of liver disease were the absence of previously known hepatitis, the presence of normal level of serum AFP (<16.5 ng/ml) and no liver mass at ultrasound examination. Written informed consent was obtained from all the patients (or from parents, in the case of children). The study protocol conformed to the ethical guidelines of the 1975 declaration of Helsinki was approved by the National Ethics Committee and the Ministry of Health of Cameroon. All the cases and controls were interviewed using a standard questionnaire that gathered information on demographic characteristics, past medical history, family history of liver disease, history of alcohol drinking, cigarette smoking, dietary history and history of blood transfusion. All the blood samples collected were aliquoted and stored at −80 °C until analyzed.

### Serological tests

#### HCV serology

The presence of antibodies against HCV (anti-HCV) was checked by the use of a third-generation enzyme immunoassay (EIA, Monolisa anti-HCV Plus version 2; Bio-Rad, Marne-La-Coquette, France). The reactivity of samples was determined as described by Njouom in 2003 [17]*.* Briefly, a ratio was calculated for each sample by dividing its optical density by the cut-off value. A positive result for anti-HCV was defined as a Monolisa ratio greater than 6 whereas all samples with a <6 ratio were scored as negative.

#### HBV serology

Different serological markers of HBV were assessed using commercial kits: hepatitis B surface antigen (HBsAg) antibody to hepatitis B core antigen (Anti-HBc), antibody to HBsAg (anti-HBs) and hepatitis e antigen (HBeAg). The presence of HBsAg was tested by enzyme-linked immunosorbent assay (ELISA) by the use of third generation reagents (Murex AgHBs version 3; DiaSorin, SPA UK BRANCH) and the presence of ant-HBc andanti-HBs were detected by enzyme immunoassay (EIA) by the using the respective commercial kits of (Monolisa; Bio-Rad, Marne-La-Coquette, France). Participants positive for HBsAg were tested for HBV “e” antigen (HBeAg) as a surrogate marker of active replication using enzyme immunoassay kit (Monolisa, Bio-Rad, Marne-La-Coquette, France). All the reactivity was determined according to the manufacturer’s instructions. Infection with HBV was defined positive when only HBsAg was detected or both HBsAg and HBeAg in the same patient.

#### HDV serology

The presence of antibodies against HDV (anti-HDV) was assessed only in HBV positive patients using commercial kits for enzyme-linked immunosorbent assay (ELISA) by the use of ETI-AB- DELTAK-2 Anti-HDV; DiaSorin, P2808). The reactivity of samples was determined according to the manufacturer instructions. . Samples with absorbance values within +/−10 % of cut-off value were retested in order to confirm the initial result. Only repeatedly reactive samples were considered positive.

### Molecular analysis

Occult hepatitis B characterized by the presence of hepatitis B virus (HBV) DNA in the serum of patients in the absence of serological markers signing active viral replication was identify in this study [18, 19] by quantification of HBV viral loads in HCC-cases negative for HBsAg. In addition we also searched for HCV RNA and quantified HCV viral loads in patients with anti-HCV antibodies to search for possible HCV occult infection. Plasma HCV-RNA and HBV-DNA levels were quantified using Abbott RealTime assay (Abbott Molecular Inc, Des Plaines, IL 60018 USA) according to manufacturer’s instructions. The lower detection limit of the assay for HCV infection was defined as viral load value greater than 12 viral RNA copies ml^−1^ (IU/mL). For HBV infection, the limit of the assay was defined as viral load value greater than 10 viral DNA copies ml^−1^(IU/mL).

### Statistical analyses

Data were presented as mean ± SD. Prevalence of HBV and HCV were compared between HCC-cases and HCC-controls. The odds ratios (ORs) were calculated to assess the risk of HCC using a conditional logistic regression analysis and confidence interval were determined. Viral loads were compared using *T* test or Mann–Whitney *U* test as appropriate following log2 transformation of the values. . The difference was considered statistically significant for *P* < 0.05. The analysis was performed using SPSS 16.0 and Prism 6.0 (GraphPad Inc, La Jolla, CA, USA) statistical softwares.

## Results

### Baseline characteristics

In all, 88 HCC-cases and 85 controls were included in this study. The mean age of HCC-cases was 46.0 ± 18.8 years (age range 8–93 years). Among the HCC-cases, 69.3 % (61/88) were men (45.8 ± 18.8 years) and 30.7 % (27/88) were women (46.5 ± 19.1 years). For the 85 controls included, 21 had diseases that need to be treated in the division of cardiology, 52 in internal medicine and 12 in the division of surgery. Every control patients was definitely diagnosed without clinical symptom of liver disease and without HCC. The mean age of the controls was 42.1 ± 16.4 years (age range: 11–82 years). Among these, 67.1 % (57/85) were men with the mean age of 41.9 ± 15.1 and 32.9 % (28/85) were women with the mean age 46.5 ± 19.1 years. The mean age between HCC-cases and controls was not significantly different. The basic demographic characteristics of patients are shown in Table 1.

**Table 1:** Demographic characteristics of patients with HCC and controls

Characteristics	HCC cases (*N* = 88)	Controls (*N* = 85)
Sex		
Male	61 (69.32 %)	59 (69.4 %)
Female	27 (30.68 %)	26 (30.6 %)
Age (Years)		
Mean age	46.01 ± 18.81	42.11 ± 16.40
Age range	8–93	11–82
Age groups		
< 20	5	5
20–39	36	36
40–59	22	22
≥ 60	25	22

*HCC* hepatocellular carcinoma

### Serological prevalence of hepatitis viruses markers in HCC patients and controls

The prevalence of HBV, HCV and HDV markers is shown in Table 2. Hepatitis B surface antigen (HBsAg) was present in 65.9 % (58/88) of the cases and in 10.6 % (9/85) of the controls. The difference between the two groups was significantly different (*P* < 0.0001). The presence of Anti-HBc positivity was found more prevalent in HCC patients than in controls patients with 82.9 and 78.8 % respectively (NS). The presence of both Anti-HBc and Anti-HBs defined as resolutive HBV was significantly (*P* < 0.05) present in controls patients compared with HCC-cases with 45.1 % vs 12.5 % respectively. Of the 58 HBsAg positive HCC-cases found, hepatitis Delta antibody (Anti-HDV) co-infection was present in 41.4 % (24/58). Only one control was co-infected with HDV infection in our study. Regarding HCV infection, the antibody against HCV (Anti-HCV) was also more frequently detected in HCC-cases compared to the controls (26.1 % (23/88) vs 3.5 % (3/85) respectively). The difference between the two groups was significantly different (*P* < 0.0001). Overall, the prevalence the serological markers for the three viruses were much higher in HCC-cases than in controls patients. Only 16 % of HCC cases (*n* = 14) were free from markers of chronic hepatitis whereas this proportion was 87 % in controls (*P* < 0.0001). Taken together, the OR associated with positivity for either HBsAg or anti-HCV was 35 (95%CI: 15.0-81.9).

**Table 2:** HBV, HCV and HDV infection in HCC patients and controls and estimates of HCC risk

Hepatitis markers	Controls		HCC cases		Unadjusted HCC risk		Adjusted HCC risks	
	No	%	No	%	OR	(95 % CI)	OR	(95 % CI)
HBV status								
HBsAg positive	9	10.6	58	65.9	16.3	7.2-37.1	141.7	11.5-1747.6
HBeAg positive	1	11.1	5	8.6	11.2	0.6-207.3	38.79	4.04-305.2
Anti-HBc positive	67	78.8	73	82.9	1.31	0.6-28	0.68	0.03-16.7
Anti-HBs positive	59	69.4	23	26.1	0.16	0.8-3.2	0.56	0.018-15.1
HCV status								
Anti-HCV positive	3	3.5	23	26.1	9.7	2.7-33.6	51.9	3.8-711.03
HDV status								
Anti-HDV positive HBV-related	1	11.1	24	41.1	5.72	0.75-39.27.	5.65	0.66-48.16
Anti-HDV positive	1	1.17	24	27.27	29.3	4.1-1231	35.65	3.66-682.16

*Note*: Adjusted analysis represents multivariable model including age gender. Abbreviations: *HCC* hepatocellular carcinoma, *HBV* hepatitis B virus, *HCV* hepatitis C virus, *HBsAg* hepatitis B surface antigen, *Anti-HBc* antibody against hepatitis B core antigen, *Anti-HBs* antibody against hepatitis B surface antigen, *Anti-HCV* antibody against hepatitis C virus, *Anti-HDV* antibody against hepatitis D virus, *CI* Confidence intervals, *OR* Odds Ratio

Dual HBV and HCV infection was observed in 5.7 % (5/88) of HCC-cases and in 1.2 % (1/85) of control and triple infection was only found in one HCC patient (2.3 %).

A small subset of patients was positive for HBV early antigen (HBeAg). These patients were much younger than the whole series (mean = 29.4 years, Range 21–36). No controls were presenting a positive serology for HBeAg. Due to the small number of positive subjects, the difference between cases and controls was, however, not reaching the degree of statistical significance (*P* = 0.059, OR = 11.2, 95%CI = 0.6-207.1).

### Hepatitis B and C viral loads in HCC patients and controls

As shown in Table 3, HBV and HCV genomes were respectively found in 93 % (54/58) and 87 % (20/23) of serologically positive HCC-cases and in 67 % (6/9) and 100 % (3/3) of the corresponding controls. HBV-DNA level were significantly higher in HBsAg-positive HCC-cases than in HBsAg-positive controls with (6.3 Log UI/mL and 5.7 Log UI/mL) respectively (*P* < 0.05). HCV-RNA levels were also higher in anti-HCV positive HCC-cases (5.5 log10) than in anti-HCV positive controls (5.2 Log 10) although the difference was not statiscally significant.

**Table 3:** Molecular analysis of HBV and HCV infections in HCC patients and controls

Variables	Controls			HCC cases		
	No	%	Mean (log10 IU/ml)	No	%	Mean (log10 IU/ml)
HBV viral load	6	67	5.7	54	93	6.3
HCV viral load	3	100	5.2	20	87	5.7

*HBV* hepatitis B virus, *HCV* hepatitis C virus

We further analyzed viral loads in HCC-HBV related co-infected with hepatitis Delta virus infection compared to those without HDV infection. The mean of HBV viral load were significantly low in patients co-infected with HDV compared with patients without HDV infection. HBV DNA loads were decreased in HDV-positive samples when compared with HBsAg (+) only (3.6 ± 1.7 vs 4.5 ± 2.1) but the difference was not statistically significant (*P* = 0.13).

Regarding occult hepatitis, 30.0 % (9/30) of HBsAg (−) patients were found positive by PCR suggesting a high rate of occult B infection (OBI) in HCC-cases in this cohort. The mean age of the 9 OBI identified was significantly higher than the mean age of patients with overt infection (59.9 ± 14.1 vs 39.04 ± 14.7 years) respectively suggesting that the kinetics of the tumor development can differ between overt and occult HBV infection. The overall proportion of patients with overt or occult B infection was thus reaching 76 % (*n* = 67/88). By contrast, no HCC case was occult for HCV in this study.

### Analysis of HBV, HCV and HDV as risk factors for HCC

The analysis of serological markers as risk factors confirmed that the risk of developing HCC was strongly associated with the three viruses in our country. The odds ratios (OR) and 95 % confidence interval (CI) for presence of HBV, HCV and HDV markers were 16.3 (7.2-37.1), 9.6 (2.8-33.6) and 29.3 (4.1-1231) respectively. Similarly, the adjusted OR with sex and age values for each infection was very high (Table 2) suggesting that, in Central Africa, the carcinogenic risk associated with HBsAg positivity is higher than the risk associated with anti-HCV sero-reactivity (Table 2). The strongest hepatocarcinogenic viral factor in Cameroonian patients was, however, the presence of an infection with HDV.

**Fig. 1:**
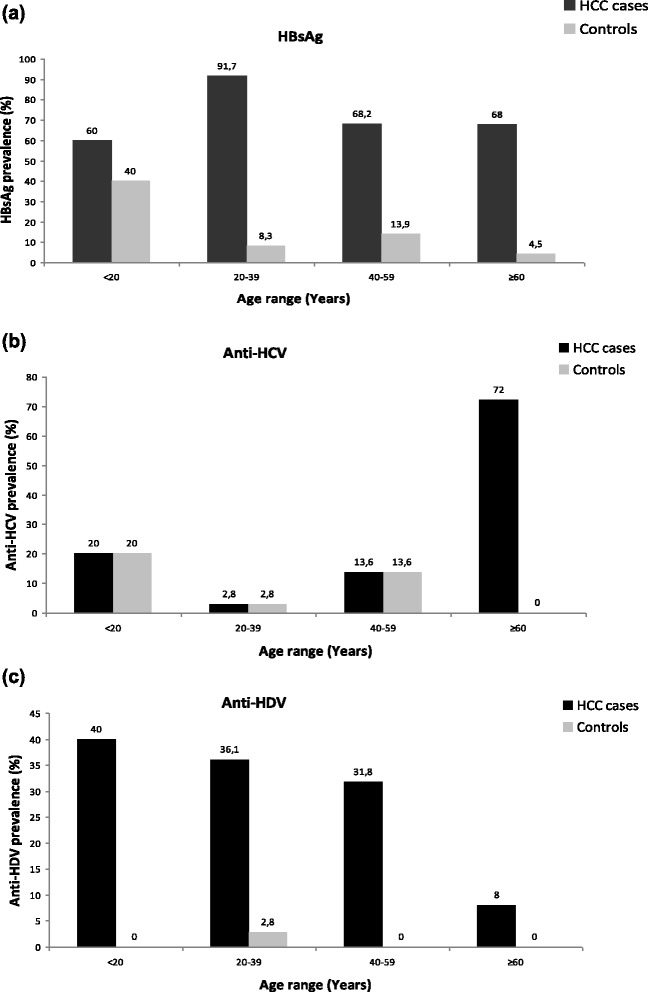
**a** Age range distribution of hepatitis surface antigen (HBsAg) in HCC cases and controls; **b** Age range distribution of antibody against hepatitis C virus (Anti-HCV) in HCC cases and controls, and (**c**): Age range distribution of antibody against hepatitis D virus (Anti-HDV) in HCC cases and controls

In order to characterize a possible stratification of the viral markers within the series examined, the association between viral hepatitis, age range and sex was also analyzed. The sex ratios of HBV, HDV and HCV positive cases were not significantly different (*P* > 0.05). By the contrast, the age distribution was significantly different (*P* < 0.05) among anti-HCV positive patients and HBsAg positive patients (64.6 ± 15.5 years vs 39.04 ± 14.7 years). The same observation was also found in patients co-infected with HDV infection. The peak of incidence of HBsAg and anti-HDV were located in the 20–39 years old subset and about 80 % of HCV-related HCC patients were in the age group ≥60 years. The liver tumor development process appears, therefore, as drastically different between both viruses in Cameroon, a situation plausibly due to the usual significant difference of age at contamination. The age distribution of the subjects analyzed is shown in Fig. 1a,b and c.

## Discussion

This is the first reported case–control study of HCC in Cameroon and to the best of our knowledge the first in Central Africa (Cameroon, Central African Republic, Chad, Gabon, Congo, Democratic Republic of Congo) an ensemble that counts 136 million inhabitants.

A close relationship between the development of HCC and older age in the settings of HBV or HCV infections has been stressed in various areas of the world [20–24].

El-Serag et al. [20]. In the current work, we observed an overall low mean age of patients (46 years) when compared to the situation prevailing in resource-rich countries where the tumor mostly occurs at an age that ranges from the late fifties to the early seventies (approximate mean age 65 years) [21–25] . Our data are, however, similar to that reported in previous studies conducted on Cameroonian HCC cases and to other studies conducted in Central Africa countries like Gabon [26]. Our finding are also in accordance with some studies conducted in West Africa countries like Nigeria [27], Gambia [28] and Ghana [29]. Our results contrast however, with those of North Africa HCC patient [23]. It seems thus, that the kinetics of tumor development is stable since decades and homogeneous throughout Sub Saharan Africa.

The high male-to-female ratio (2.3) observed in this Cameroonian cohort of HCC cases is similar to those reported from the East (2.4), the West (2.2) and the South (2.3) of Africa [14] and in the literature. This may be due in part from higher prevalence of HBV and HCV infections among men and/or to the fact that males usually have history of drinking alcohol [30–33] .

In the present study, all three viruses responsible for chronic hepatitis were strongly associated with HCC development in Cameroon. Our results show that HBV is the predominant risk factor associated with HCC development in our country; as we found, it is involved in 65 % of cases with overt infection and in 75 % of cases when both overt and occult B infections were regrouped. Furthermore, almost all of our patients had a serological marker of previous HBV infection. The high prevalence of HBV infection was consistently reported in various groups of patients in Cameroon: 10.1 % in blood donors [12], 20.4 % in pregnant women [10], 23.6 % in health care workers [11] and 33 % in the Bantus enrolled in Central, South, North West and East part of the country [13]. Our findings regarding HBV prevalence in HCC is in keeping with data reported for decades in Gabon (40.5 %) and many studies conducted in West African countries like Niger (73 %), Senegal and Mali (63 %), Gambia (60 %) and Nigeria 61 % [26, 34–36]. This observation confirms that HBV infection is still the most important risk factor of HCC development in Sub Saharan Africa and that Central Africa does not differ from other sub Saharan regions.

We observed the presence of Anti-HCV in 26 % of the HCC-cases. Similar levels were found in different studies conducted in Sub-Saharan Africa [34, 36]. Our results contrast, however, with those of the study conducted in 2008 by Perret et al. on hepatitis B and C chronics patients from Gabon where the prevalence of the two viruses were equal and many study conducted in HCC-cases of the North of Africa were it is HCV infection which is generally more prevalent. In Cameroon, as in many other countries, anti-HCV carriers represent a birth cohort (>50 years old) for which the disease-associated burden will progressively decreases as this generation will pass away.

Much less is known about HDV in Sub-Saharan Africa. In this study, a high proportion of patients co-infected with Hepatitis Delta virus was also detected in HCC cases (41.1 %) compared to control individuals (1.1 %). The present result is consistent with previous studies conducted on non HCC patients with liver diseases in Cameroon [9, 13], in many Sub-Saharan countries and the literature [29]. By contrast, our findings were markedly different from that reported in Nigerian HCC cases [37] where it was reported no HDV co-infection both in HCC cases and controls.

The impact of active replication is known to be associated with an increased risk of HCC development [38, 39]. In the present work, the mean of HBV and HCV viral loads were significantly high in HCC patients compared to control patients. Regarding patients co-infected with HDV, the means of HBV viral load were significantly low in patients co-infected with HDV compared with patients without HDV infection (15328.4 IU/ml vs 261264.1 IU/ml) respectively. This result indicates as reported by previous studies the inhibition of HBV replication in HDV infection [9, 40]. We observed above 16-fold, 10-fold and 29-fold odds ratios increase for HCC risk in HBV, HCV and HDV infections respectively. Our data indicate that HBV and HCV infections are involved in the vast majority of HCC-cases observed in Cameroon. We detected at least one viral marker in around 85 % of cases. This result indicates that most Cameroonians HCC cases are resulting from a hepatotropic virus-related chronic liver disease. Similarly to what was reported in Cameroon and elsewhere, we observed that tumor onset is drastically different (25 years apart) between HBV- and HCV-infected patients. This observation suggests either that it takes longer to HCV to result in a HCC or that HCV infection occuring later in life (through contact with inappropriate health practices for example) as compared to vertical or early horizontal HBV transmission, kinetics of both tumorigenic processes are grossly similar. Overall, both vertical transmission and early horizontal infection for HBV [41, 42] coupled to parenteral exposition in the 1980s for HCV [43–46] explain the spread of both viruses in Cameroon and provide the epidemiological bases of liver tumor development.

In this study, approximately 15 % of the HCC patients were negative for HBV and HCV markers. Our finding suggests that there is thus a significant involvement of non-viral factors in the incidence of HCC development in Cameroon. In many African and Asian countries, HCC has been associated with chronic exposure to Aflatoxin B1, a mycotoxin known to be present in Cameroon staple-food such as maize [47]. There are some limitations in this study that should be considered. First, we used only anti-Delta Ab to validate the association between HDV infection and HCC; second, HBV DNA and HCV RNA were not searched in negatives HBV and HCV controls. Molecular analysis will better assess the interplay between HDV infection and estimate the real implication of HBV and HCV infections in the development of HCC.

Beside environment, lifestyle risk factors, including prolonged abuse of alcohol or cigarette smoking, both highly prevalent in Cameroon, are known to increase liver cancer risk [1, 5]. All these factors were not evaluated in this study and their contributory role as causative agents of HCC in Cameroon is still poorly documented. There is therefore an urgent need for further studies aiming to evaluate their possible involvement of non-viral co-carcinogenic factors acting alone or in association with the viruses on HCC development.

## Conclusion

In summary, this study provides for the first time a landscape of the major viral risk factors associated with HCC development in Cameroon and in Central Africa. Our results show that patients co-infected with HDV or mono-infected with HBV are at very high risk to develop HCC. We consider that in absence of an easy access to novel antiviral compounds, effective preventive measures aiming to control HBV transmission (vaccination at birth, better hygiene) are of paramount importance to reduce the pool of Cameroonians citizens at risk and significantly curb down the future HCC incidence in Central Africa including Cameroon. Among all individuals at risk, HDV-infected represent the most worrying subpopulation due to the almost complete lack of efficient antiviral compounds active on HDV and to the remarkably high relative risk of HCC associated with this viral infection. A national epidemiological survey aiming to identify groups at risk to be HDV-infected and a closer surveillance of hepatitis delta among women in reproductive age should be undertaken now in Cameroon.

## Abbreviations

Anti-HCV: Antibody to HCV
Anti-HDV: Antibody to HDV
CI: Confidence interval
HBsAg: Hepatitis B surface antigen
HBV: Hepatitis B virus
HCC: Hepatocellular carcinoma
HCV: Hepatitis C virus
HDV: Hepatitis D virus
OR: Odd ratio
